# Supplementary material for: Patterns of Intron Gain and Loss in Fungi
Source: PLoS Biol. 2004 Nov 30;2(12):e422. doi: 10.1371/journal.pbio.0020422 (PMC532390; doi:10.1371/journal.pbio.0020422)
Supplement: Table S1 — Also available at http://genes.mit.edu/NielsenEtAl/. (4.3 MB ZIP). [file pbio.0020422.st001.zip › NielsenEtAl/html/289.html]

AN3855.1.NCU01275.1.MG02439.1.FG05919.1


```
 CLUSTAL W (1.82) Multiple Sequence Alignments - Introns Inserted


Sequence 1: NCU01275.1	333 aa
Sequence 2: MG02439.1	390 aa
Sequence 3: FG05919.1	350 aa
Sequence 4: AN3855.1	346 aa
Alignment Length: 395 aa
Number Identitical Residues: 170 aa
Alignment Score (without introns) 8641


MG02439.1 	MSAKGPITTPLTQLLGIKHPIVLAGMARVSGGKLAAAVSNAGGLGVIGGFMYTPDQLREI
NCU01275.1	-------------------------MARVSGGPLAAAVSNAGGLGVIGGFMYTPDQLRSI
FG05919.1 	MSPPGPITTPLTSLLGIKHPIVLAGMARVSGGRLAAAVSNAGGLGIIGGFQYTPKQLREI
AN3855.1  	MS----ITTPLTKLLNIQSPVLLAGMAKASGAPLAAAVSNAGGLGVIGGLGYTPEQMNEM
          	 :     ::. :.  . . .   :.**:.**. ************:***: ***.*:..:

MG02439.1 	IAEMKANFDTPDLPFGVDLALPQVGGNARKTNHDYTGGKLDELIDITIESGAKLFVSAVG
NCU01275.1	IAEMKANFSRPDLPFGVDLALPQIGGSARKTNHDYTGGKLDELIDITIESGARLFVSAVG
FG05919.1 	LAEMKANFSRPDLPFGVDLALPQIGGNARKTNHDYTGGKLDELVDIIIDSGAKLFVSAVG
AN3855.1  	LTELKSLLKDKSLPFGVDLALPQIGGNARKTNHDYTGGKLDELIDVIISHGAKLFVSAVG
          	::*:*: :.  .***********:**.****************:*: *. **:*******

MG02439.1 	VPPKHVIDRLHGAGIYVMNMVGHPKHA~VKALDLGVDLVCPQGGEGGGHTGDIANSVLIP
NCU01275.1	VPPKRIIDKLHANGILVMNMVGHPKHA~VKALQLGVDMVCAQGGEGGGHTGDVANSVLIP
FG05919.1 	VPPPEVIKRFHDKGILVMNMVGHPKHA0TKALELGVDMICAQGGEGGGHTGDVANSILIP
AN3855.1  	VPPAHVIKRLHEAGILIMNMVGAPKHA~EKALKAGVDIICAQGGEGGGHTGDIPFSVLIP
          	*** .:*.::*  ** :***** ****  ***. ***::*.***********:. *:***

MG02439.1 	AVVDVARRYRPKMLG-----GKQHAIVVAAGGIADGRGLASSLMQGAAGVWVGTRFVASV
NCU01275.1	SVVDVARMFKPTMLNKVLGTTDQPALVVAAGGIADGRGLASSLMQGAAGVWVGTRFVASK
FG05919.1 	AVADIASKYHPPLLK------GLPALVLAAGGISSGRSLASSLMQGASGVWVGTRFVASE
AN3855.1  	AVVDTVQKYKSPLTG-------QTPLVVAAGGVNDGRSLAAALTLGASGVWVGTRFLATE
          	:*.* .  ::. :           .:*:****: .**.**::*  **:********:*: 

MG02439.1 	ESNSSDEHKQAVVDCNPEDTERTLVLSGRPLRLKVNDYIRKWHARPDRIKELCDKGIVPI
NCU01275.1	EASCSDQHKEAVVTCGFQDTERTLVLSGRPLRMKTNQYIAKWHAQPEKIAELTEKGIVPI
FG05919.1 	EASCSEEHKKAVVSCGYEDTQRTLVISGRPLRMKTNEYIQKWHDQPDKIKELCDKGVVPL
AN3855.1  	ESGASKLHKQAVINAQYGETKRTLVVSGRPLRLLPNDYIKEWEKKPEEIARLTSQGIVPM
          	*:..*. **:**: .   :*:****:******:  *:** :*. :*:.* .* .:*:**:

MG02439.1 	EHDFENGTENGGEIDLPHLMGQVAGNIKKVEPAGDIVRSMVAEAVEMIKLG1YEGWLRE2
NCU01275.1	EWDMDQ----GNEIDPPHLMGQVAGLVKKVQPAGEIVEEMVQEAVEQLKLG~-------~
FG05919.1 	EYDFEQ----GNDFDLPHLMGQVAGAITKVQPAGEIVDEMVKEAVEMLKLG~-------~
AN3855.1  	EHDFNN----DKEVDLPFLMGDVSGIIKDIKPAGVVLKEMVEQAADVLKRT~-------~
          	* *:::    . :.* *.***:*:* :..::*** :: .** :*.: :*           

MG02439.1 	HLALLEAINVDNNSRGCSGVYTKPSGQTPG2LPVSAENG
NCU01275.1	------------------QAYLN-GGRTSK~L-------
FG05919.1 	------------------GTYLT---AMPR~L-------
AN3855.1  	------------------AALAS--GPASK~L-------
          	                   .  .  .  .  *
```
